# Therapie des oralen Lichen planus – eine aktuelle Übersicht

**DOI:** 10.1007/s00105-025-05540-x

**Published:** 2025-07-15

**Authors:** Khava Abdusalamova, Farzan Solimani, Margitta Worm

**Affiliations:** Klinik für Dermatologie, Venerologie und Allergologie, Charité – Universitätsmedizin Berlin, Luisenstr. 2, 10117 Berlin, Deutschland

**Keywords:** Erosiver Lichen planus, Behandlung, T‑Zell-Erkrankung, Glukokortikoide, Retinoide, Erosive lichen planus, Drug therapy, T‑cell diseases, Glucocorticoids, Retinoids

## Abstract

Der Lichen planus mucosus (MLP) ist eine chronisch rezidivierende, entzündliche T‑Zell-Erkrankung, die Schleimhäute befallen kann und zusammen mit dem kutanen Lichen planus (CLP) und dem Lichen planopilaris (LPP) zu den Kreisformen der Lichen planus(LP)-Erkrankungen gehört. MLP kann sowohl die Mundschleimhaut (oraler Lichen planus [OLP]) als auch die Genitalschleimhaut betreffen. Patienten mit OLP können unterschiedliche Schweregrade aufweisen. Insbesondere Patienten, die von einer erosiven/ulzerativen Form betroffen sind, stellen in der Regel eine therapeutische Herausforderung dar, da I) es bisher keine von der FDA (Food and Drug Administration)/EMA (European Medicines Agency) zugelassenen Medikamente gibt und II) die Krankheit häufig eine Therapieresistenz aufweist. Kürzlich veröffentlichte Studien zur Immunpathogenese beschreiben eine überwiegende Rolle der durch Interferon‑γ ausgelösten Entzündung, doch viele Aspekte der Krankheit sind noch unbekannt. Dementsprechend stehen bislang primär symptomatische Therapien zur Verfügung. Neben topischen Glukokortikoiden stellen Retinoide in topischer und systemischer Form sowie systemische Glukokortikoide die etablierten Erstlinientherapien dar. Eine Vielzahl von Zweit- und Drittlinientherapien zeigt, dass bisher keine standardmäßige Behandlung existiert. Bei den neuen Therapien sind Januskinaseinhibitoren sowie monoklonale Antikörper hervorzuheben, die zukünftig das Therapiespektrum für OLP erweitern könnten. Hierfür sind jedoch prospektive, placebokontrollierte Studien zukünftig notwendig. Auch alternative und ergänzende Behandlungen wie pflanzliche Therapien, Lichttherapie sowie „platelet-rich plasma“ (PRP) und „injectable platelet-rich fibrin“ (i-PRF), werden in der Literatur beschrieben. In diesem Beitrag diskutieren wir die aktuellen therapeutischen Optionen für diese schwer zu behandelnde Krankheit.

## Abstract (en)

Mucosal lichen planus (MLP) is a chronic, recurrent, inflammatory T‑cell disorder that can affect mucous membranes and, together with cutaneous lichen planus (CLP) and lichen planopilaris (LPP), belongs to the variants of lichen planus (LP) diseases. MLP can affect both the oral mucosa (oral lichen planus, OLP) and the genital mucosa. Patients with OLP can have varying degrees of severity. In particular, patients affected by an erosive/ulcerative form usually pose a therapeutic challenge because 1) there are currently no US Food and Drug Administration(FDA)/European Medicines Agency (EMA)-approved drugs and 2) the disease is often treatment resistant. Recently published studies on immunopathogenesis describe a predominant role of interferon-γ-induced inflammation, but many aspects of the disease are still unknown. Accordingly, primarily symptomatic therapies are currently available. In addition to topical glucocorticoids, retinoids in topical and systemic form and systemic glucocorticoids are established first-line therapies. A wide range of second- and third-line therapies show that no standard treatment that there is still o standard treatment. Among the new therapies, Janus kinase inhibitors and monoclonal antibodies should be emphasized, as they could expand the therapeutic spectrum for OLP in the future. However, prospective, placebo-controlled studies are needed for this in the future. Alternative and complementary treatments such as herbal therapies, phototherapy, platelet-rich plasma (PRP) and injectable platelet-rich fibrin (i-PRF) are also described in the literature. In this article, we discuss the current therapeutic options for this difficult-to-treat disease.

Der Lichen planus (LP) ist eine chronisch rezidivierende, entzündliche T‑Zell-Erkrankung, der die Haut, Schleimhäute und Adnexe betreffen kann. Es werden 3 Hauptformen von LP unterschieden: der kutane Lichen planus (CLP), der mukosale Lichen planus (MLP) und der Lichen planopilaris (LPP). Während der CLP einen häufig selbstlimitierenden Verlauf zeigen kann, stellt der MLP und hier insbesondere der orale Lichen planus (OLP) eine therapeutische Herausforderung dar [1]. Ursprünglich wurde OLP in 6 verschiedene Formen eingeteilt (retikulär, papulär, plaqueartig, erosiv, atrophisch und bullös), aber gewöhnlich wird er in 2 Formen unterteilt: retikulär oder erosiv/ulzerativ (Abb. 1; [2]). Das klinische Bild des OLP erlaubt die Unterscheidung von unter anderem 2 Varianten (Abb. 1). Eine Variante ist der retikuläre OLP, bei dem eine charakteristische weißliche Streifung sichtbar ist, die auch als Wickham-Streifung bezeichnet wird. Die Wickham-Streifung wird durch eine Hyperproliferation des Stratum granulosum der Epidermis ausgelöst. Der ulzerative OLP kann häufig an der Basis des retikulären OLP auftreten und entsprechend häufig sind Mischformen zu beobachten. Der retikuläre OLP verläuft meist mit milden Beschwerden und kann in den meisten Fällen mit topischen Maßnahmen erfolgreich behandelt werden. Die erosive/ulzerative Variante ist im Gegenteil eine aggressive Erkrankung, die in der Regel behandlungsresistent ist. Eine Sonderform ist die desquamative Gingivitis mit Befall der Zahnfleischschleimhaut, was oft zu chronischer Parodontitis führen kann [3]. Außerdem ist die erosive bzw. ulzerierende Form häufig besonders schmerzhaft und kann durch Probleme bei der Nahrungsaufnahme zu einer erheblichen Beeinträchtigung der Lebensqualität der Patienten führen [4]. Diese Form des OLP bedarf meist einer systemischen immunsuppressiven Therapie. Unbehandelt kann der OLP zu Strikturen und fibrotischen Veränderungen sowie zum Befall weiterer Strukturen bzw. Organe wie Nasopharynx, Pharynx, Larynx und Ösophagus führen [5]. Dieser letzte Punkt unterstreicht die Notwendigkeit, die Krankheit bei diesen Patienten erfolgreich zu kontrollieren.

**Abb. 1:**
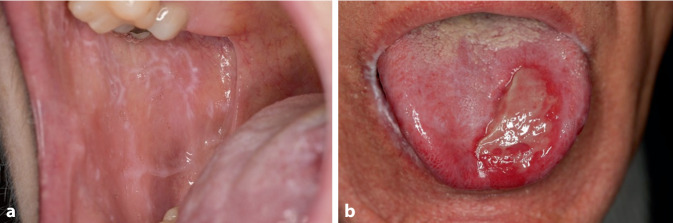
Retikulärer oraler Lichen planus (OLP) der Wangenschleimhaut (**a**) vs. erosiver/ulzerierender OLP der Zunge (**b**)

Mit einer Prävalenz von 0,89 % in der allgemeinen Bevölkerung ist der OLP keine seltene Erkrankung [6]. Frauen und Patienten >40 Jahre sind dabei häufiger betroffen [6]. Hinzu kommt, dass der OLP durch die chronische Inflammation die Entstehung von Plattenepithelkarzinomen begünstigen kann. Dabei wird das Entartungsrisiko gemäß der Literatur auf 1,43 % geschätzt [7]. Eine angemessene Behandlung und regelmäßige Follow-ups der betroffenen Patienten sind daher von großer Bedeutung.

Die genaue Pathogenese des OLP ist derzeit noch nicht vollständig geklärt. Es konnte bereits gezeigt werden, dass die Erkrankung auf einer T‑Zell-vermittelten Immunreaktion gegen orale Keratinozyten beruht, die zu deren Apoptose führt [8]. Dabei scheinen insbesondere IFN(Interferon)-γ-abhängige Mechanismen bei der Immunpathogenese eine zentrale Rolle zu spielen [9, 10]. Entsprechend stehen bisher nur symptomatische Therapien mit antientzündlicher, immunmodulatorischer oder immunsuppressiver Wirkung zur Verfügung. Verschiedene Therapeutika werden zur Behandlung des OLP eingesetzt (Abb. 2), wobei Kortikosteroide, Retinoide und Ciclosporin als Erstlinientherapien derzeit empfohlen werden. Es steht jedoch ein breites Spektrum von weiteren Therapiemöglichkeiten zur Verfügung.

**Abb. 2:**
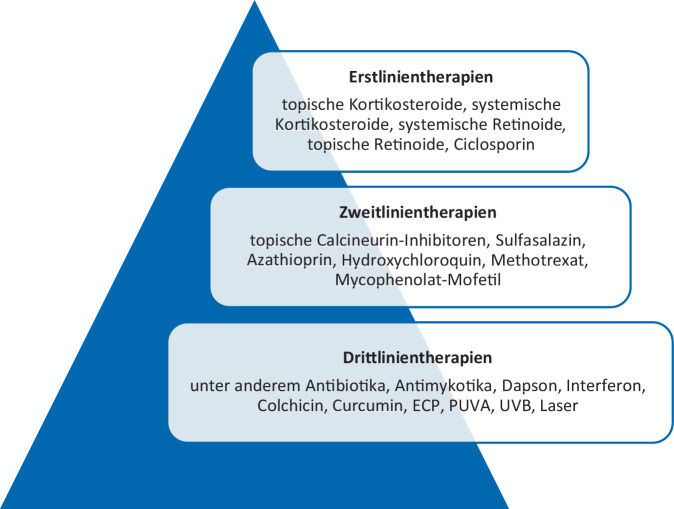
Therapieformen für oralen Lichen planus (OLP) gemäß der europäischen S1-Leitlinie für Lichen planus (LP). *ECP* extrakorporale Photopherese, *PUVA* Psoralen plus UVA [11]

In diesem Beitrag geben wir einen aktuellen Überblick über die verschiedenen Erst- und Zweitlinientherapien sowie eine Auswahl an Drittlinientherapien und besprechen Literatur zum aktuellen Forschungsstand. Hierfür wurde eine PubMed-Suche mit dem Suchbegriff *Lichen planus* und dem Filter *systematic review* durchgeführt (200 Suchergebnisse), um systematische Reviews zum Thema Behandlung von OLP zu identifizieren. Darüber hinaus wurde nach den einzelnen Therapieformen und dem Begriff *oraler Lichen planus* gesucht.

Zur Evaluierung des Therapieerfolges wurden in den unterschiedlichen Studien verschiedene Parameter herangezogen. Gängige Parameter waren Schmerz und klinische Scores wie Reticular Erythematous Ulceration (REU) oder Oral Disease Severity Score (ODSS).

## Kortikosteroide

Topische Kortikosteroide sind bis heute die etablierte Erstlinientherapie des OLP [11]. Sie stehen in verschiedenen Formulierungen wie Mundspüllösungen, Cremes, Haftsalben, Sprays oder Gelen zur Verfügung. Die am häufigsten verwendeten Wirkstoffe sind Clobetasolpropionat, Triamcinolon, Betamethason, Fluocinonid, Fluticason, Dexamethason und Prednisolon [11]. Welche Darreichungsform und welcher Wirkstoff am wirksamsten sind, ist derzeit nicht systematisch untersucht worden. In der Regel werden die topischen Kortikosteroide von den Patienten gut vertragen. Dennoch kann es zu Nebenwirkungen wie Kandidose, Trockenheit der Mundschleimhaut, Atrophie, Geschmacksstörungen, Dyspepsie, Übelkeit und auch zu einer systemischen Absorption kommen. Trotz des weit verbreiteten Einsatzes von topischen Steroiden bei OLP zeigt unsere Analyse der publizierten Literatur, dass die Wirksamkeit topischer Kortikosteroide zur Behandlung des OLP nur schwach durch klinische Studien belegt ist. Cheng et al. verglichen in einer Übersichtsarbeit mit 473 Patienten verschiedene lokale und systemische Therapieformen miteinander und kamen zu dem Schluss, dass keine Therapie überlegen war, auch nicht topische Kortikosteroide [12]. Lodi et al. untersuchten in ihrer Cochrane-Analyse (2020) von 35 Arbeiten mit insgesamt 1474 Patienten die Wirksamkeit von topischen und systemischen Kortikosteroiden und konnten im Vergleich zur Placebogruppe zeigen, dass primär die Schmerzlinderung bei Kortikosteroiden wahrscheinlicher war [13]. In Bezug auf Parameter wie klinische Besserung und Nebenwirkungen waren die Ergebnisse nicht eindeutig. Die Autoren weisen darauf hin, dass die Aussagkraft dieser Ergebnisse aufgrund der geringen Anzahl von Studien und Patienten als begrenzt einzustufen ist [13]. Intraläsional applizierte Kortikosteroide zählen ebenfalls zur Gruppe der topischen Kortikosteroide. Triamcinolonacetonid, Hydrocortison, Dexamethason oder Methylprednisolon werden bei insbesondere erosivem OLP direkt in die Läsionen injiziert [11].

Schließlich werden auch systemische Kortikosteroide als Erstlinientherapie bei OLP eingesetzt. Zu den häufig verwendeten Wirkstoffen zählen Methylprednisolon und Prednison (30–80 mg/Tag) [11]. Systemische Kortikosteroide werden primär bei schweren und therapieresistenten Verläufen verwendet. Eine langfristige Anwendung sollte aufgrund ihrer systemischen Nebenwirkungen vermieden und durch steroidsparende Medikamente ersetzt werden. Drei von 35 Studien zur Steroidwirksamkeit untersuchten die Wirkung von systemischen Kortikosteroiden, davon eine Studie die Wirkung einer oralen Stoßtherapie mit Betamethason im Vergleich zu einem topischen Steroid (Triamcinolonacetonid) [13]. Beide Therapien waren vergleichbar effektiv [14]. Insgesamt ist die Steroidtherapie eine wichtige Säule der OLP-Therapie. Insbesondere Haftsalben und Mundspüllösungen werden regelmäßig eingesetzt. Bei schweren Formen kann eine systemische Steroidstoßtherapie als initiale systemische Therapie notwendig sein.

## Steroidsparende Therapien

Die hier aufgeführten Medikamente werden in der europäischen Leitlinie als Erst- und Zweitlinientherapien genannt [11]. In Deutschland ist keine der besprochenen Substanzen für die Behandlung des OLP zugelassen und die Anwendung kann nur als Off-label-Therapie eingesetzt werden.

### Retinoide

Topische und systemische Retinoide werden in der europäischen Leitlinie zur Behandlung des OLP als Erstlinientherapie aufgeführt [11]. Zu den gängigen Wirkstoffen gehören Acitretin und Isotretinoin [11]. Bei dieser Gruppe handelt es sich um Vitamin-A-Derivate, die immunmodulierend und antiinflammatorisch wirken.

Die Wirksamkeit von Retinoiden wurde jedoch nur in einer begrenzten Anzahl von Studien untersucht. In einer Übersichtsarbeit wurden 28 Studien eingeschlossen, von denen nur eine die Wirksamkeit von topischen Retinoiden bei 10 Patienten betrachtete [15].

In einer Übersichtsarbeit von Sandhu et al. wurde unter anderem die Wirksamkeit von systemischem Etretinat im Vergleich zu Placebo bei 28 Patienten analysiert [16]. Die Behandlung mit dem systemischen Retinoid war gegenüber Placebo überlegen, es kam jedoch zu verschiedenen Nebenwirkungen wie Trockenheit der Haut und Schleimhäute, Keratokonjunktivitis, Exanthem, Kopfschmerzen, Pruritus und Haarverlust [17]. Insgesamt basiert die Wirksamkeit von Retinoiden auf Studien, die im Rahmen von CLP durchgeführt wurden. Die Wirksamkeit von Retinoiden scheint interindividuelle Unterschiede zu haben. Auch die Nebenwirkungen sollten berücksichtigt werden. Bei der Verwendung von Retinoiden muss deren Teratogenität stets berücksichtigt und mit den Patienten sorgfältig besprochen werden. Bei Frauen im fortpflanzungsfähigen Alter ist daher eine zuverlässige Empfängnisverhütung bis 3 Jahre nach Absetzen von Retinoiden zu gewährleisten [18].

### Tacrolimus und Pimecrolimus

Calcineurininhibitoren in ihrer topischen Formulierung können eine Alternative zur chronischen topischen Steroidanwendung darstellen [11]. Die Wirkung von Calcineurininhibitoren beruht auf der Hemmung der T‑Zell-Aktivität durch Reduktion unter anderem der Interleukin-2-Produktion. Orales Ciclosporin wird mit 3–10 mg/kg/Tag bei den Erstlinientherapien aufgeführt, während topisches Tacrolimus und Pimecrolimus zu den Zweitlinientherapien zählen. In einer kürzlich erschienenen Übersichtsarbeit mit 1114 Patienten wurden topische Immunmodulatoren mit anderen Therapieformen des OLP verglichen. Die Autoren zeigen, dass Tacrolimus und Pimecrolimus in ihrer klinischen Wirkung und bezüglich der Symptomlinderung vergleichbar mit topischen Kortikosteroiden waren [15].

Zu einem ähnlichen Ergebnis kamen Sun et al. in ihrer Übersichtsarbeit mit 21 Studien und 965 Patienten. Auch die kurzfristige Gabe von Tacrolimus führte häufiger zu Nebenwirkungen als bei topischen Kortikosteroiden [19]. Die Autoren empfehlen daher topische Calcineurininhibitoren bei Unwirksamkeit von Kortikosteroiden als alternative Behandlung in Erwägung zu ziehen. Eine weitere Übersichtsarbeit von 9 randomisierten kontrollierten Studien von Su et al. zeigt, dass kein signifikanter Unterschied zwischen Tacrolimus und Kortikosteroiden in Bezug auf das klinische Ansprechen und die Rezidivhäufigkeit besteht [20]. Auch in dieser Übersichtsarbeit wurde das häufigere Auftreten von allerdings zumeist milden Nebenwirkungen mit Tacrolimus berichtet [20]. Insgesamt zeigen die Daten, dass topische Calcineurininhibitoren bezüglich ihrer Wirksamkeit vergleichbar mit topischen Kortikosteroiden sind, wobei häufiger mit Nebenwirkungen wie brennenden Schmerzen, Sialorrhö, Xerostomie und Geschmacksveränderungen zu rechnen ist [20].

### Ciclosporin

Ciclosporin zeigte gemischte Ergebnisse. Einerseits zeigte es im Vergleich zu Placebo eine höhere Effizienz bei der Kontrolle der Symptome, andererseits aber auch eine geringere Wirksamkeit im Vergleich zu Steroiden. Außerdem war die Anwendung von Ciclosporin mit dem Auftreten zahlreicher Nebenwirkungen assoziiert: brennende Schmerzen, gastrointestinale Beschwerden, Brustspannen, Schwindel, Juckreiz, Schwellung der Lippen, Blutungen und Petechien [15]. In Anbetracht des höheren Risikoprofils und der nicht eindeutigen Wirksamkeit von Cyclosporin sollte dieses Medikament nach Ansicht der Autoren nur dann eingesetzt werden, wenn sich andere Behandlungsmöglichkeiten als unwirksam erwiesen haben oder nicht verfügbar sind.

### Sulfasalazin

Sulfasalazin ist ein Prodrug, das v. a. für die Behandlung der chronisch entzündlichen Darmerkrankungen eingesetzt wird. Bei OLP wurde die Wirksamkeit von topischem Sulfasalazin (30 mg in 5 ml destilliertem Wasser 3‑mal täglich für 4 Wochen) in einer offenen, nicht placebokontrollierten Studie bei 21 therapieresistenten Patienten auf topische/systemische Kortikosteroide untersucht; 81 % der Patienten sprachen auf die Behandlung an und 57 % erreichten eine Abnahme der Läsionsgröße >50 % [21].

### Azathioprin

Azathioprin ist ein Purinanalogon und zählt zu den Immunsuppressiva. Es wird nach Organtransplantation, bei verschiedenen Autoimmunerkrankungen und bei chronisch entzündlichen Darmerkrankungen eingesetzt. In einer kleinen Fallstudie (*n* = 9) wurde die Wirkung von Azathioprin (50 mg 2‑mal täglich oral [ca. 2 mg/kg pro Tag], für 3 bis 7 Monate) bei OLP untersucht. Bei 8/9 Patienten kam es zu einem sehr guten bis guten Ansprechen auf die Behandlung [22].

### Hydroxychloroquin

Hydroxychloroquin (HCQ) ist ein Antimalariamittel, das v. a. als Basistherapeutikum zur Behandlung des Lupus erythematodes eingesetzt wird. In einer kürzlich erschienenen Übersichtsarbeit mit 390 Patienten aus 11 Studien wurde die Wirksamkeit von Antimalariamitteln bei OLP untersucht. In 5 Studien, die Schmerz als Parameter erfassten, kam es zu einer Schmerzlinderung unter Hydroxychloroquin [23]. Währenddessen berichteten 6 weitere Studien über eine objektive klinische Verbesserung der Erkrankung [23]. Zu den häufigsten berichteten Nebenwirkungen zählten Sehstörungen, Magenbeschwerden, Exanthem, Übelkeit, Kopfschmerzen, Hautpigmentierung und erhöhte Nierenfunktion [23]. Auch in einer kürzlich von uns veröffentlichten Studie zeigte HCQ bei Patienten mit OLP und CLP sowohl eine gute Wirksamkeit als auch eine gute Verträglichkeit [24]. Im Vergleich zu anderen steroidsparenden Medikamenten (Retinoide, Azathioprin, Cyclosporin) wirkt HCQ eher immunmodulatorisch als immunsuppressiv und hat somit ein besseres Sicherheitsprofil, was auch zu einer besseren Patientenakzeptanz führt.

### Methotrexat

Methotrexat ist ein Folsäureantagonist und wirkt immunsuppressiv und zytostatisch. Es wurden 18 Patienten mit erosivem OLP im Rahmen einer prospektiven offenen Studie mit Methotrexat 15 mg wöchentlich für 12 Wochen oral behandelt. Bei 15 von 18 Patienten kam es zu einem partiellen oder kompletten Ansprechen [25]. Chauhan et al. konnten ebenfalls in ihrer prospektiven Beobachtungsstudie die Wirksamkeit von Methotrexat allein (0,3 mg/kg 1‑mal wöchentlich für 16 Wochen) oder in Kombination mit einem topischen Kortikosteroid bei moderatem bis schwerem OLP nachweisen [26].

### Mycophenolat-Mofetil

Mycophenolat-Mofetil (MMF) ist ein Immunsuppressivum, das die Proliferation von T‑ und B‑Lymphozyten hemmt. Bisher konnte die gute Wirksamkeit von MMF bei OLP nur in Fallberichten (*n* = 1) (2000 mg/Tag) und retrospektiven Beobachtungsstudien (*n* = 10) (500 mg bis 2 g täglich für 2 Monate) nachgewiesen werden [27, 28]. Eine randomisierte klinische Studie mit 27 Patienten zur Untersuchung der Wirksamkeit von topischem MMF (MMF 2 % läsional 2‑mal täglich für 4 Wochen) konnte zeigen, dass MMF effektiv Schmerzen und Größe von Ulzerationen verringert [29].

## Drittlinientherapien

In der Literatur wurden verschiedene Medikamente und Verfahren zur Behandlung des OLP in Einzelfällen oder kleineren Fallserien berichtet. Im Einzelfall können diese für die Behandlung in Ausnahmefällen bei Nichtansprechen auf die Erst- oder Zweitlinientherapie erwogen werden. Hierzu gehören unter anderem Antibiotika, Antimykotika, Dapson, Interferon, Colchicin oder auch die extrakorporale Photopherese (ECP), PUVA(Psoralen plus UVA)-Therapie, UVA- und UVB-Therapie sowie Lasertherapie [11]. Eine Auswahl dieser Drittlinientherapien sowie neuere Therapien werden im Folgenden näher erläutert.

### Monoklonale Antikörper

Für den OLP gibt es bislang keinen zugelassenen therapeutischen Antikörper. Verschiedene monoklonale Antikörper (mAbs) wurden bislang bei wenigen Patienten mit therapieresistentem OLP angewendet. Hierzu zählen TNF(Tumornekrosefaktor)-α-Hemmer (Adalimumab, Etanercept, Infliximab), IL(Interleukin)-17-Inhibitoren (Secukinumab), IL-12/23-Inhibitoren (Ustekinumab) und IL-23-Inhibitoren (Guselkumab, Tildrakizumab) sowie T‑Zell-Modulatoren (Alefacept) und Rituximab [30]. Es gibt Hinweise darauf, dass diese biologischen Therapien eine klinische Verbesserung oder komplette Heilung bei OLP (*n* = 22) erzielen können [30]. In einer ersten randomisierten, doppelblinden, placebokontrollierten Phase-II-Studie mit Secukinumab bei 111 Patienten, von denen ein Drittel an einem MLP litt, konnte gezeigt werden, dass der IL-17-Inhibitor gut vertragen wird und zu einer numerischen Verbesserung des Investigator Global Assessment (IGA) bei OLP und Lichen planopilaris führt [31]. Eine Verbesserung des klinischen Scores REU zeigte sich jedoch nicht. Die begrenzte Studienlage lässt jedoch keine systematische Analyse der Wirksamkeit und Sicherheit dieser mAbs bei OLP zu. Insgesamt zeigen diese Ergebnisse, dass einige Patienten mit OLP eine IL-17-Signatur aufweisen, die ein therapeutisches Ziel sein kann. Andererseits scheint es angesichts der aktuellen Datenlage unwahrscheinlich, dass die IL-17-Hemmung bei allen Patienten wirksam ist.

### Januskinaseinhibitoren

Erste vielversprechende Ergebnisse zur Anwendung von Januskinase(JAK)-Inhibitoren bei OLP wurden kürzlich in einzelnen Fallberichten publiziert. Diese Medikamentenklasse ist bei OLP sehr vielversprechend, da sie die Blockade von Interferon‑γ ermöglicht. Die Wirkung von JAK-Inhibitoren beruht auf der Blockade des JAK-STAT(„signal transducers and activators of transcription“)-Signalweges über Hemmung der verschiedenen JAK-Proteine [32]. Abduelmula et al. fassten in ihrer kürzlich publizierten Arbeit Fallberichte und Studien zum Einsatz von JAK-Inhibitoren bei LP (6/56 Patienten mit OLP) zusammen und kamen zu folgenden Ergebnissen für Tofacitinib (*n* = 30), Baricitinib (*n* = 16), Ruxolitinib (*n* = 12) und Upadacitinib (*n* = 2): Es kam bei 25 % der Patienten (*n* = 4/16) unter Baricitinib, bei 10 % (*n* = 3/30) unter Tofacitinib, bei 16,7 % (*n* = 2/12) unter Ruxolitinib und 100 % (2/2) unter Upadacitinib zur kompletten Remission der Erkrankung [33]. Ein weiterer JAK1-Inhibitor, der in einem Fallbericht als gut wirksam bei OLP beschrieben wurde, ist Abrocitinib [34]. Zusätzlich wurde in einer kürzlich erschienenen Fallstudie der Effekt des TYK2-Inhibitors Deucravacitinib bei 3 Patienten mit OLP untersucht, wobei es bei allen 3 Patienten zur deutlichen Verbesserung der Beschwerden kam [35]. Derzeit wird weltweit eine Reihe von placebokontrollierten klinischen Studien durchgeführt, um die Wirksamkeit dieser Medikamente zu testen. Erste Ergebnisse aus einem Phase-II-Trial für Baricitinib bei CLP bestätigen die gute Wirksamkeit dieser Medikamente bei LP-Erkrankungen [36] (clinicaltrials.gov).

## Alternative und ergänzende Therapien

### Low-level-Lasertherapie

Die Low-level-Lasertherapie (LLLT) stellt eine nichtmedikamentöse Behandlungsoption für OLP dar. Sie wird auch als Photobiomodulation bezeichnet und soll regenerative, analgetische und antiinflammatorische Effekte haben. Mehrere Übersichtsarbeiten konnten die positive Wirkung von LLLT auf OLP nachweisen, wobei LLLT sowohl effektiv als auch nebenwirkungslos war [37–39]. Akram et al. verglichen LLLT (Diodenlaser, In:Ga:Al:P-Laser) mit Kortikosteroiden und waren sich unschlüssig, ob LLLT tatsächlich wirksamer als Kortikosteroide ist [40]. In einer von 5 Studien schnitten nämlich LLLT und Kortikosteroide gleich gut ab, während in 3 Studien topische Kortikosteroide signifikant überlegen waren. In der letzten Studie dagegen wurde eine signifikante Überlegenheit der LLLT gegenüber Kortikosteroiden festgestellt [40]. Insgesamt scheint diese Therapie in Bezug auf Wirksamkeit und logistische Durchführbarkeit nur für einzelne Patienten infrage zu kommen, die nicht auf die übliche Medikation ansprechen.

### Photodynamische Therapie

Die photodynamische Therapie (PDT) ist eine weitere Form der Lichttherapie, bei der unterschiedliche Photosensibilisatoren durch sichtbares Licht entsprechender Wellenlänge aktiviert werden. Aktuell wird die PDT als neue Behandlungsoption bei OLP erprobt. In einer Übersichtsarbeit aus 16 Studien von 2020 verglichen He et al. PDT mit Kortikosteroiden und konnten zeigen, dass PDT eine ähnliche Wirksamkeit wie topische Kortikosteroide hatte [41]. Es kamen Diodenlaser (3 Studien), Xenonlampe (eine Studie), Halbleiterlaser (eine Studie), Metallhalogenlampe (eine Studie) und lichtemittierende Diode (LED) (4 Studien) zum Einsatz. Die Mehrheit der Patienten hatte keine oder nur leichte Beschwerden wie milde brennende Schmerzen während der Behandlung. Sie schlussfolgerten deshalb, dass PDT eine effektive Behandlungsoption für OLP darstellen kann und bei Wirkungslosigkeit oder Kontraindikation von Kortikosteroiden in Erwägung gezogen werden sollte [41]. Auch andere Übersichtsarbeiten kamen zu ähnlichen Ergebnissen und wiesen gleichzeitig auf die Notwendigkeit weiterer qualitativ hochwertiger Studien hin [42–49].

### Pflanzliche und natürliche Therapien

Die unten aufgeführten Therapieoptionen können eine gute Ergänzung und Erhaltungstherapie für Patienten mit minimaler Krankheitsaktivität oder Remission sein, reichen aber möglicherweise nicht aus, um die Krankheit allein zu kontrollieren.

#### *Aloe vera*

*Aloe vera* ist ein pflanzliches Heilmittel mit antientzündlichen, antimikrobiellen und wundheilungsfördernden Eigenschaften. Es ist als Mundspüllösung oder Mundgel erhältlich. In einer Übersichtsarbeit mit insgesamt 217 Patienten von 2017 verglichen die Autoren *Aloe vera* mit Placebo oder Kortikosteroiden (Triamcinolonacetonid) und konnten eine leichte Evidenz für eine bessere Wirksamkeit von *Aloe vera* im Vergleich mit Placebo und eine ähnliche Wirksamkeit im Vergleich mit Kortikosteroiden (Triamcinolonacetonid) feststellen [50]. Die Metaanalyse von 3 Studien hingegen ergab eine Unterlegenheit von *Aloe vera* im Vergleich mit der Kontrollgruppe [50]. *Aloe vera* kann eine sinnvolle Ergänzung sein und dazu beitragen, den Einsatz von Steroiden zu reduzieren.

#### Hyaluronsäure

Bei Hyaluronsäure (HA) handelt es sich um ein im Bindegewebe vorkommendes Glykosaminoglykan. Waingade et al. stellen die Ergebnisse von 7 Studien vor, die HA topisch als Gel oder Mundspülung mit Placebo, Kortikosteroiden oder Calcineurininhibitoren verglichen [51]. Es fanden sich keine statistisch signifikanten Ergebnisse für den Vergleich von HA mit Kontrollgruppen bei hoher Heterogenität der eingeschlossenen Studien. Aus qualitativer Sicht kamen die Autoren zu der Schlussfolgerung, dass HA aufgrund fehlender Nebenwirkungen als alternative Therapie bei OLP empfohlen werden kann [51].

#### Curcumin

*Curcuma longa*, auch bekannt als Kurkuma, enthält den Wirkstoff Curcumin, der antientzündliche, immunmodulatorische und antioxidative Eigenschaften aufweist. In 3 Übersichtsarbeiten wurde die Wirksamkeit speziell von Curcumin bei OLP untersucht. Eine Übersichtsarbeit von Moayeri et al. ergab, dass Curcumin keinen signifikanten Einfluss auf Erythem, Läsionsgröße oder Schmerz bei OLP hat [52]. White et al. hingegen konnten nachweisen, dass topisches Curcumin Schmerzen, Brennen und das klinische Bild von OLP im Vergleich zur Baseline verbessert [53]. Auch Lv et al. konnten bei Behandlung mit Curcumin im Vergleich zur Baseline eine statistisch signifikante Besserung des klinischen Bildes von OLP feststellen. Im Vergleich mit Kortikosteroiden konnten keine statistisch signifikanten Unterschiede gefunden werden [54].

#### Nahrungsergänzungsmittel

Unter den Nahrungsergänzungsmitteln war der Effekt von Vitamin D auf die Behandlung von OLP von Interesse. Vitamin D werden immunmodulatorische und antientzündliche Eigenschaften zugeschrieben. Die systematische Analyse von Saeed et al. (2022) untersuchte den Effekt von Vitamin D auf OLP. Eine signifikante Verbesserung der Symptome und des klinischen Bildes, der Läsionsgröße bzw. -schwere wurde beschrieben [55]. Die Autoren kamen daher zu der Schlussfolgerung, Vitamin-D-Supplementation als ergänzende Therapie bei OLP zu erwägen [55].

#### „Platelet-rich-plasma“ und „injectable-platelet-rich fibrin“

„Platelet-rich-plasma“ (PRP) und „injectable platelet-rich fibrin“ (i-PRF) sind Produkte, die aus körpereigenem Blut gewonnen werden und Plasma mit einer erhöhten Konzentration von Blutplättchen und Wachstumsfaktoren enthalten. Diese Produkte werden in die betroffenen Bereiche injiziert, sie wirken dort immunmodulatorisch und antiinflammatorisch und fördern die Wundheilung und Regeneration des Gewebes [56]. Mehrere Studien haben die Anwendung von PRP bzw. i‑PRF bei OLP untersucht und positive Ergebnisse hinsichtlich der Symptomverbesserung gezeigt [56–58].

## Diskussion

Die Behandlung des OLP stellt bis heute eine therapeutische Herausforderung dar. Topische Therapien sind Mittel der ersten Wahl, wobei topische Kortikosteroide am häufigsten eingesetzt werden und am besten untersucht sind. Des Weiteren stehen steroidsparende Therapien wie Calcineurininhibitoren, Retinoide und konventionelle Immunsuppressiva wie Azathioprin und Methotrexat zur Verfügung. Insbesondere bei der ulzerativen Form der OLP ist eine Kombinationstherapie mit topischen und systemischen Medikamenten erforderlich, um die Entzündung erfolgreich zu unterdrücken. Neben diesen gängigen Therapien wurden verschiedene alternative Behandlungsoptionen zur Behandlung des OLP in der Literatur beschrieben. Die photodynamische Therapie sowie die Low-level-Lasertherapie zeigen zur Behandlung des OLP positive Ergebnisse, insbesondere bei Patienten, die nicht auf Kortikosteroide ansprechen oder bei denen diese kontraindiziert sind. Darüber hinaus haben auch pflanzliche und natürliche Therapien wie *Aloe vera*, Hyaluronsäure, Curcumin und PRP bzw. i‑PRF sowie Nahrungsergänzungsmittel wie Vitamin D einen positiven Einfluss auf die Symptome von OLP und könnten als ergänzende Therapieoptionen erwogen werden.

Neuere Therapien wie mAbs und JAK-Inhibitoren sind zur Behandlung von OLP wirksam und könnten eine wirksame Alternative zu den derzeitigen steroidsparenden Immunsuppressiva darstellen. Diese Therapien sind jedoch nicht von der FDA (Food and Drug Administration)/EMA (European Medicines Agency) zugelassen, und es sind kontrollierte prospektive Studien erforderlich. Es bleibt von zentraler Bedeutung, die Patienten erfolgreich zu behandeln und die Entzündungsaktivität zu minimieren. Chronischer OLP könnte

- zu einem Verlust der Organfunktion aufgrund von Fibrose,
- zu chronischer Parodontitis und Zahnverlust,
- zur Begünstigung der Entwicklung von Plattenepithelkarzinomen

führen. Um die Behandlung der Patienten zu gewährleisten, sind individueller und interdisziplinärer Behandlungsansatz unter Einbeziehung des Patienten und Berücksichtigung von Nebenerkrankungen, medikamentöser Therapie und Zahnstatus von großer Bedeutung. Zusätzlich sollte das Ansprechen der Erkrankung regelmäßig standardisiert evaluiert werden und eine entsprechende Anpassung des Therapieplans erfolgen. Aufgrund des Malignomrisikos sind regelmäßige klinische Kontrollen notwendig und bioptische Untersuchungen werden bei unklaren Läsionen empfohlen. Es ist zu bedenken, dass mukosale Plattenepithelkarzinome eine höhere Metastasierungsrate aufweisen als kutane Plattenepithelkarzinome [59]. Bezüglich der unterschiedlichen klinischen Manifestationen des OLP (retikulär vs. erosiv) gibt es bislang in Bezug auf die klinische Wirksamkeit der bis heute eingesetzten medikamentösen Therapien gemäß unserer Literaturrecherche keine Daten. Jedoch ist zu vermuten, dass aufgrund pathophysiologischer Unterschiede und insbesondere der T‑Zell-Expressionsprofile der betroffenen Schleimhaut möglicherweise Unterschiede bestehen. Es ist jedoch anzumerken, dass retikuläre Formen häufig mit topischen Therapien behandelt werden können (mit anfänglicher täglicher Anwendung und anschließender proaktiver Therapie). Im Gegensatz dazu erfordern erosive Formen in der Regel eine Kombinationstherapie aus topischen und systemischen Medikamenten. Um neue therapeutische Ansätze und zielgerichtete Therapien zu entwickeln, besteht Forschungsbedarf in der Aufklärung der genauen pathophysiologischen Grundlagen der Erkrankung. Zusätzlich werden standardisierte, placebokontrollierte Studien mit ausreichender Fallzahl zum Vergleich der verschiedenen Therapieformen untereinander benötigt. Die Heterogenität der aktuellen Studien und das hohe Risiko für studienbedingte Verzerrungen lassen nur begrenzt evidenzbasierte Schlussfolgerungen zu.

## Fazit für die Praxis

- Das breite Spektrum der therapeutischen Optionen für die Behandlung von oralem Lichen planus (OLP) zeigt, dass es bisher keine Standardtherapie gibt.
- Die derzeitigen Erkenntnisse deuten darauf hin, dass die Standardtherapie auf topischen Steroiden basiert, die in der Regel zusammen mit systemischen steroidsparenden Medikamenten verabreicht werden.
- Eine positive Wirkung von Medikamenten wie JAK(Januskinase)-Inhibitoren oder monoklonalen Antikörpern (mAbs) ist vielversprechend, muss aber in klinisch kontrollierten Studien mit ausreichender Patientenzahl und standardisierten Ergebnismessungen auf ihre Wirksamkeit hin untersucht werden.
- Bestimmte natürliche Therapien haben eine entzündungshemmende Wirkung und könnten in bestimmten Situationen eine entzündungshemmende unterstützende Wirkung entfalten.
- Andere Therapien wie die photodynamische Therapie, die Low-level-Lasertherapie und PRP („platelet-rich plasma“)/i-PRF („injectable platelet-rich fibrin“) könnten in behandlungsresistenten Einzelfällen versucht werden.
- Es besteht nach wie vor ein großer Forschungsbedarf zur Beantwortung grundlegender Fragen der OLP-Immunpathogenese, womit hoffentlich die Behandlung dieser stark belastenden Krankheit ermöglicht wird.

## References

[1] Solimani F, Forchhammer S, Schloegl A, Ghoreschi K, Meier K (2021) Lichen planus – ein Klinikleitfaden. J Dtsch Dermatol Ges 19(6):864–883 34139075 10.1111/ddg.14565_g

[2] Andreasen JO (1968) Oral lichen planus. 1. A clinical evaluation of 115 cases. Oral Surg Oral Med Oral Pathol 25(1):31–42 5235654 10.1016/0030-4220(68)90194-1

[3] Sanadi RM, Khandekar PD, Chaudhari SR, Javali MA, Gurav NU (2023) Association of periodontal disease with oral lichen planus: A systematic review and meta analysis. J Oral Maxillofac Pathol 27(1):173–180 37234328 10.4103/jomfp.jomfp_178_22 PMC10207185

[4] Fiocco Z, Kupf S, Patzak L, Kämmerer T, Pumnea T, French LE et al (2021) Quality of Life and Psychopathology in Lichen Planus: A Neglected Disease Burden. Acta Derm Venereol 101(12):adv619 34698356 10.2340/actadv.v101.442 PMC9472096

[5] Macken JH, Senusi A, O’Toole EA, Caley M, Rognoni E, Fortune F (2024) Erosive lichen planus: an unmet disease burden. Front Med 17(11):1457667 10.3389/fmed.2024.1457667 PMC11524830 39484200

[6] Li C, Tang X, Zheng X, Ge S, Wen H, Lin X et al (2020) Global Prevalence and Incidence Estimates of Oral Lichen Planus: A Systematic Review and Meta-analysis. JAMA Dermatol 156(2):172–181 31895418 10.1001/jamadermatol.2019.3797 PMC6990670

[7] González-Moles MÁ, Ramos-García P (2024) An Evidence-Based Update on the Potential for Malignancy of Oral Lichen Planus and Related Conditions: A Systematic Review and Meta-Analysis. Cancers 16(3):608 38339358 10.3390/cancers16030608 PMC10854587

[8] Louisy A, Humbert E, Samimi M (2024) Oral Lichen Planus: An Update on Diagnosis and Management. Am J Clin Dermatol 25(1):35–53 37713153 10.1007/s40257-023-00814-3

[9] Pietschke K, Holstein J, Meier K, Schäfer I, Müller-Hermelink E, Gonzalez-Menendez I et al (2021) The inflammation in cutaneous lichen planus is dominated by IFN‑ϒ and IL-21‑A basis for therapeutic JAK 1 inhibition. Exp Dermatol 30(2):262–270 33113249 10.1111/exd.14226

[10] Shao S, Tsoi LC, Sarkar MK, Xing X, Xue K, Uppala R et al (2019) IFN‑γ enhances cell-mediated cytotoxicity against keratinocytes via JAK 2/STAT1 in lichen planus. Sci Transl Med 11(511):eaav7561 31554739 10.1126/scitranslmed.aav7561 PMC7285657

[11] Ioannides D, Vakirlis E, Kemeny L, Marinovic B, Massone C, Murphy R et al (2020) European S1 guidelines on the management of lichen planus: a cooperation of the European Dermatology Forum with the European Academy of Dermatology and Venereology. J Eur Acad Dermatol Venereol 34(7):1403–1414 32678513 10.1111/jdv.16464

[12] Cheng S, Kirtschig G, Cooper S, Thornhill M, Leonardi-Bee J, Murphy R (2012) Interventions for erosive lichen planus affecting mucosal sites. Cochrane Database Syst Rev 2012(2):CD8092 22336835 10.1002/14651858.CD008092.pub2 PMC10794897

[13] Lodi G, Manfredi M, Mercadante V, Murphy R, Carrozzo M (2020) Interventions for treating oral lichen planus: corticosteroid therapies. Cochrane Database Syst Rev 2(2):CD1168 32108333 10.1002/14651858.CD001168.pub3 PMC7047223

[14] Malhotra AK, Khaitan BK, Sethuraman G, Sharma VK (2008) Betamethasone oral mini-pulse therapy compared with topical triamcinolone acetonide (0.1 %) paste in oral lichen planus: A randomized comparative study. J Am Acad Dermatol 58(4):596–602 18158199 10.1016/j.jaad.2007.11.022

[15] da Silva EL, de Lima TB, Rados PV, Visioli F (2021) Efficacy of topical non-steroidal immunomodulators in the treatment of oral lichen planus: a systematic review and meta-analysis. Clin Oral Investig 25(9):5149–5169 34342763 10.1007/s00784-021-04072-7

[16] Sandhu S, Klein BA, Al-Hadlaq M, Chirravur P, Bajonaid A, Xu Y et al (2022) Oral lichen planus: comparative efficacy and treatment costs—a systematic review. Bmc Oral Health 22(1):161 35524296 10.1186/s12903-022-02168-4 PMC9074269

[17] Hersle K, Mobacken H, Sloberg K, Thilander H (1982) Severe oral lichen planus: treatment with an aromatic retinoid (etretinate). Br J Dermatol 106(1):77–80 7037037 10.1111/j.1365-2133.1982.tb00904.x

[18] Soprano DR, Soprano KJ (1995) Retinoids as teratogens. Annu Rev Nutr 15:111–132 8527214 10.1146/annurev.nu.15.070195.000551

[19] Sun SL, Liu JJ, Zhong B, Wang JK, Jin X, Xu H et al (2019) Topical calcineurin inhibitors in the treatment of oral lichen planus: a systematic review and meta-analysis. Br J Dermatol 181(6):1166–1176 30903622 10.1111/bjd.17898

[20] Su Z, Hu J, Cheng B, Tao X (2022) Efficacy and safety of topical administration of tacrolimus in oral lichen planus: An updated systematic review and meta-analysis of randomized controlled trials. J Oral Pathol Med 51(1):63–73 34133803 10.1111/jop.13217

[21] Jeong SH, Na HS, Park SH, Ahn YW, Chung J (2016) Topical sulfasalazine for unresponsive oral lichen planus. Quintessence Int 47(4):319–327 26504904 10.3290/j.qi.a34974

[22] Verma KK, Mittal R, Manchanda Y (2001) Azathioprine for the treatment of severe erosive oral and generalized lichen planus. Acta Derm Venereol 81(5):378–379 11800155 10.1080/000155501317140197

[23] Tillero R, González-Serrano J, Caponio VCA, Serrano J, Hernández G, López-Pintor RM (2024) Efficacy of antimalarials in oral lichen planus: A systematic review. Oral Dis 30(7):4098–4112 38720635 10.1111/odi.14975

[24] Abdusalamova K, Worm M, Solimani F (2025) Hydroxychloroquine is safe and efficacious in oral lichen planus: data from a large outpatient cohort. Arch Dermatol Res 317(1):711 40232322 10.1007/s00403-025-04226-7 PMC12000255

[25] Lajevardi V, Ghodsi SZ, Hallaji Z, Shafiei Z, Aghazadeh N, Akbari Z (2016) Treatment of erosive oral lichen planus with methotrexate. J Dtsch Dermatol Ges 14(3):286–293 26972194 10.1111/ddg.12636

[26] Chauhan P, De D, Handa S, Narang T, Saikia UN (2018) A prospective observational study to compare efficacy of topical triamcinolone acetonide 0.1 % oral paste, oral methotrexate, and a combination of topical triamcinolone acetonide 0.1 % and oral methotrexate in moderate to severe oral lichen planus. Dermatol Ther 31(1) 10.1111/dth.12563 29124831

[27] Wee JS, Shirlaw PJ, Challacombe SJ, Setterfield JF (2012) Efficacy of mycophenolate mofetil in severe mucocutaneous lichen planus: a retrospective review of 10 patients. Br J Dermatol 167(1):36–43 22309851 10.1111/j.1365-2133.2012.10882.x

[28] Dalmau J, Puig L, Roé E, Peramiquel L, Campos M, Alomar A (2007) Successful treatment of oral erosive lichen planus with mycophenolate mofetil. J Eur Acad Dermatol Venereol 21(2):259–260 17243970 10.1111/j.1468-3083.2006.01832.x

[29] Samiee N, Taghavi Zenuz A, Mehdipour M, Shokri J (2020) Treatment of oral lichen planus with mucoadhesive mycophenolate mofetil patch: A randomized clinical trial. Clin Exp Dent Res 6(5):506–511 32592335 10.1002/cre2.302 PMC7545225

[30] Didona D, Caposiena Caro RD, Sequeira SAM, Solimani F, Hertl M (2022) Therapeutic strategies for oral lichen planus: State of the art and new insights. Front Med (9):997190 10.3389/fmed.2022.997190 PMC9578567 36267615

[31] Passeron T, Reinhardt M, Ehst B, Weiss J, Sluzevich J, Sticherling M et al (2024) Secukinumab in adult patients with lichen planus: efficacy and safety results from the randomized placebo-controlled proof-of-concept PRELUDE study. Br J Dermatol 191(5):680–690 38735684 10.1093/bjd/ljae181

[32] Solimani F, Hilke FJ, Ghoreschi K (2019) Pharmakologie der Januskinaseinhibitoren [Pharmacology of Janus kinase inhibitors. Hautarzt 70(12):934–941 31740978 10.1007/s00105-019-04509-x

[33] Abduelmula A, Bagit A, Mufti A, Yeung KCY, Yeung J (2023) The Use of Janus Kinase Inhibitors for Lichen Planus: An Evidence-Based Review. J Cutan Med Surg 27(3):271–276 36815857 10.1177/12034754231156100 PMC10291104

[34] Solimani F, Mesas-Fernández A, Dilling A, Nast A, Hilke FJ, Ghoreschi FC et al (2023) The Janus kinase 1 inhibitor abrocitinib for the treatment of oral lichen planus. J Eur Acad Dermatol Venereol 10.1111/jdv.19069 36974430

[35] Stolte KN, Mesas-Fernández A, Meier K, Klein EK, Dommisch H, Ghoreschi K et al (2024) TYK2 inhibition with deucravacitinib ameliorates erosive oral lichen planus. Exp Dermatol 33(4):e15080 38628035 10.1111/exd.15080

[36] Hwang AS, Kechter JA, Do TH, Hughes AN, Zhang N, Li X et al (2024) Rapid response of lichen planus to baricitinib associated with suppression of cytotoxic CXCL13+CD8+ T cells. J Clin Invest 135(2):e179436 39541169 10.1172/JCI179436 PMC11735091

[37] Ruiz Roca JA, López Jornet P, Gómez García FJ, Aroca MP (2022) Effect of Photobiomodulation on Atrophic-Erosive Clinical Forms of Oral Lichen Planus: A Systematic Review. Dent J 10(12):221 10.3390/dj10120221 PMC9776719 36547037

[38] Al-Maweri SA, Kalakonda B, Al-Soneidar WA, Al-Shamiri HM, Alakhali MS, Alaizari N (2017) Efficacy of low-level laser therapy in management of symptomatic oral lichen planus: a systematic review. Lasers Med Sci 32(6):1429–1437 28536905 10.1007/s10103-017-2233-7

[39] Mahuli SA, Rai A, Shree P, Ul Haque Z, Mahuli AV (2024) Efficacy of photobiomodulation in the management of oral Lichen Planus in comparison to topical corticosteroids: Systematic review, meta-analysis, and GRADE-based assessment of certainty of evidence. J Stomatol Oral Maxillofac Surg 125(5S2):101798 38387618 10.1016/j.jormas.2024.101798

[40] Akram Z, Abduljabbar T, Vohra F, Javed F (2018) Efficacy of low-level laser therapy compared to steroid therapy in the treatment of oral lichen planus: A systematic review. J Oral Pathol Med 47(1):11–17 28766756 10.1111/jop.12619

[41] He Y, Deng J, Zhao Y, Tao H, Dan H, Xu H et al (2020) Efficacy evaluation of photodynamic therapy for oral lichen planus: a systematic review and meta-analysis. BMC Oral Health 20(1):302 33148217 10.1186/s12903-020-01260-x PMC7640434

[42] Al-Maweri SA, Ashraf S, Kalakonda B, Halboub E, Petro W, AlAizari NA (2018) Efficacy of photodynamic therapy in the treatment of symptomatic oral lichen planus: A systematic review. J Oral Pathol Med 47(4):326–332 29350426 10.1111/jop.12684

[43] Waingade M, Medikeri RS, Rathod P (2022) Effectiveness of methylene blue photosensitizers compared to that of corticosteroids in the management of oral lichen planus: a systematic review and meta-analysis. J Dent Anesth Pain Med 22(3):175–186 35693351 10.17245/jdapm.2022.22.3.175 PMC9171335

[44] Gulzar MA, Gul N, Alvi FD, Khattak YR, Hasan US, Haneef MB et al (2023) Comparison of photodynamic therapy and corticosteroid therapy in management of oral lichen planus: A systematic review of randomized controlled trials. Photodiagnosis Photodyn Ther 44:103747 37567329 10.1016/j.pdpdt.2023.103747

[45] Akram Z, Javed F, Hosein M, Al-Qahtani MA, Alshehri F, Alzahrani AI et al (2018) Photodynamic therapy in the treatment of symptomatic oral lichen planus: A systematic review. Photodermatol Photoimmunol Photomed 34(3):167–174 29223131 10.1111/phpp.12371

[46] Nagi R, Muthukrishnan A, Rakesh N (2023) Effectiveness of photodynamic therapy (PDT) in the management of symptomatic oral lichen planus—A systematic review. J Oral Biol Craniofac Res 13(2):353–359. (Epub 2023 Mar 12. Erratum in: J Oral Biol Craniofac Res. 2024 Jul–Aug;14(4):353–354.) 36941903 10.1016/j.jobcr.2023.03.003 PMC10023948

[47] Hanna R, Dalvi S, Tomov G, Hopper C, Rebaudi F, Rebaudi AL et al (2023) Emerging potential of phototherapy in management of symptomatic oral lichen planus: A systematic review of randomised controlled clinical trials. J Biophotonics 16(7):e202300046 37017292 10.1002/jbio.202300046

[48] Wang B, Fan J, Wang L, Chai L (2021) Photobiomodulation Therapy/Photodynamic Therapy Versus Steroid Therapy for Oral Lichen Planus: A Systematic Review and Meta-Analysis. Photobiomodul Photomed Laser Surg 39(3):145–154 33601953 10.1089/photob.2020.4930

[49] Hoseinpour Jajarm H, Asadi R, Bardideh E, Shafaee H, Khazaei Y et al (2018) The effects of photodynamic and low-level laser therapy for treatment of oral lichen planus—A systematic review and meta-analysis. Photodiagnosis Photodyn Ther 23:254–260 30006319 10.1016/j.pdpdt.2018.07.001

[50] Ali S, Wahbi W (2017) The efficacy of aloe vera in management of oral lichen planus: a systematic review and meta-analysis. Oral Dis 23(7):913–918 28029732 10.1111/odi.12631

[51] Waingade M, Medikeri RS, Gaikwad S (2022) Effectiveness of hyaluronic acid in the management of oral lichen planus: a systematic review and meta-analysis. J Dent Anesth Pain Med 22(6):405–417 36601134 10.17245/jdapm.2022.22.6.405 PMC9763825

[52] Moayeri H, Rajabi A, Mohammadi M, Moghaddam SB (2024) Effects of Curcumin on the treatment of oral lichen planus symptoms: a systematic review and meta-analysis study. BMC Oral Health 24(1):104 38233780 10.1186/s12903-024-03873-y PMC10795217

[53] White CM, Chamberlin K, Eisenberg E (2019) Curcumin, a turmeric extract, for oral lichen planus: A systematic review. Oral Dis 25(3):720–725 30614166 10.1111/odi.13034

[54] Lv KJ, Chen TC, Wang GH, Yao YN, Yao H (2019) Clinical safety and efficacy of curcumin use for oral lichen planus: a systematic review. J Dermatolog Treat 30(6):605–611 30388912 10.1080/09546634.2018.1543849

[55] Saeed S, Choudhury P, Ahmad SA, Alam T, Panigrahi R, Aziz S et al (2022) Vitamin D in the Treatment of Oral Lichen Planus: A Systematic Review. Biomedicines 10(11):2964 36428531 10.3390/biomedicines10112964 PMC9687323

[56] Sriram S, Hasan S, Alqarni A, Alam T, Kaleem SM, Aziz S et al (2023) Efficacy of Platelet-Rich Plasma Therapy in Oral Lichen Planus: A Systematic Review. Med 59(4):746 10.3390/medicina59040746 PMC10146996 37109704

[57] Maddheshiya N, Srivastava A, Rastogi V, Shekhar A, Sah N, Kumar A (2023) Platelet-rich plasma protein as a therapeutic regimen for oral lichen planus: An evidence-based systematic review. Natl J Maxillofac Surg 14(1):22–26 37273445 10.4103/njms.njms_504_21 PMC10235744

[58] Zhang Y, Mao C, Zhu J, Yu W, Wang Z, Wang Y et al (2023) Effect of platelet concentrates for pain and symptom management in oral lichen planus: an evidence-based systematic review. Bmc Oral Health 23(1):594 37626383 10.1186/s12903-023-03296-1 PMC10463801

[59] Bugshan A, Farooq I (2020) Oral squamous cell carcinoma: metastasis, potentially associated malignant disorders, etiology and recent advancements in diagnosis. F1000Res 9:229 32399208 10.12688/f1000research.22941.1 PMC7194458

